# Synergistic antibacterial mechanism of the *Lactobacillus crispatus* surface layer protein and nisin on *Staphylococcus saprophyticus*

**DOI:** 10.1038/s41598-017-00303-8

**Published:** 2017-03-21

**Authors:** Zhilan Sun, Pengpeng Li, Fang Liu, Huan Bian, Daoying Wang, Xiaomeng Wang, Ye Zou, Chong Sun, Weimin Xu

**Affiliations:** 1 Institute of Agricultural Products Processing, Jiangsu Academy of Agricultural Sciences, Nanjing, 210014 PR China; 2 Key Laboratory of Meat Processing and Quality Control, Ministry of Education, Nanjing Agricultural University, Nanjing, 210095 PR China

## Abstract

SlpB, a surface layer protein isolated from *Lactobacillus crispatus*, has the potential to enhance the antimicrobial activity of nisin. Previous research indicated that, when combined with nisin, SlpB acted synergistically to inhibit *Staphylococcus saprophyticus* growth, thus extending the shelf life of chicken meat. In order to understand how SlpB enhances the antibacterial activity of nisin, electron microscopy, confocal laser scanning microscopy, flow cytometry and transmembrane electrical potential analysis were used to study cell wall organization and cell membrane integrity. No remarkable bacteriolytic effects were observed, indicating that cell death could not be attributed to cell lysis, although SlpB caused dramatic modifications of cell wall, thereby altering cell shape. The combination of SlpB and nisin also induced the release of ATP or UV-absorbing materials, as well as sudden dissipation of the transmembrane electrical potential by compromising membrane integrity. Considering that SlpB led to structural disorganization of the cell wall, and nisin access is enhanced to form a stable pore, cell death is a predictable outcome. SlpB significantly enhanced the effect of nisin at half of the minimum inhibitory concentration, which resulted in cell death by destroying the cell wall and cell membrane, therefore providing a new, feasible approach in food preservation.

## Introduction

*Staphylococcus saprophyticus* are the dominant microorganism in cheese (14%), raw milk (12%), and dry sausages (10.9%)^1^. The existence of nosocomial and urinary tract infections related to *S. saprophyticus* has raised questions about the safety of *S. saprophyticus* ^2^. Moreover, von Eiff *et al.* observed the occurrence and pathogenic potential of *S. saprophyticus*, suggesting that it could be an emerging pathogen^3^, which indicates that strategies to control *S. saprophyticus* in foods are necessary. Meat and related products are among the leading vehicles for *S. saprophyticus* transmission^4^ and the rise of *S. saprophyticus* contamination necessitates the development of new antimicrobial agents^5^. Due to concerns regarding negative consumer perception of chemical preservatives, recent studies have focused on the antimicrobial efficacy of “natural” compounds^6^.

Nisin, a Class I bacteriocin produced by *Lactococcus lactis* subsp. *lactis*, is the only bacteriocin that is generally recognized as safe for food preservation in dairy and meat products^7^. However, due to its relatively narrow antibacterial spectrum, low solubility, and instability at physiological pH, it acts as a poor antibacterial when used alone *in vivo* ^8, 9^. In order to offset this effect, nisin can be combined with new antibacterial agents to synergistically control food spoilage and certain food pathogens (e.g., *Staphylococcus* or *Salmonella*)^10, 11^. S-layer proteins are monomolecular crystalline arrays that consist of a single homogeneous protein or glycoprotein that range from 40–200 kDa^12^. The biological functions of *Lactobacillus* S-layer proteins are poorly understood, but some S-layer proteins mediate bacterial adherence to host cells/extracellular matrix proteins or have protective functions^13, 14^. Previously, a new murein hydrolase S-layer protein (SA) from *Lactobacillus acidophilus* was found to act against the cell wall (CW) of *Salmonella enterica* serovar Newport, thereby exhibiting a potential application in food preservation^15^. However, the effect of SA on whole cells from Gram-positive bacteria was not observed. The role of murein hydrolases as S-layer proteins has previously been described from *Bacillus anthracis* and *L. acidophilus*; however, there is little information on the mechanism of S-layer proteins in spoilage or against pathogenic bacteria. Additionally, S-layer protein SA acts synergistically with nisin and allow reduction of the levels of nisin and control of bacterial growth, although the biological relevance of this combination remains to be elucidated^16^. In contrast, the antimicrobial mechanism of nisin has been extensively studied and is well-documented^17^. Nisin binds to lipid II and prevents peptidoglycan network synthesis and nisin-lipid II complexes assemble to form a stable pore in the cell membrane of target cells^18^. Acosta *et al.* postulated that either SA enhances nisin access to the cell membrane by enabling travel across the CW or nisin causes a sudden ion-nonspecific dissipation of the proton motive force required to enhance SA murein hydrolase activity^16^. However, the synergistic mechanism of the SA-nisin combination remains to be elucidated. Aside from *L. acidophilus*, currently no other *Lactobacillus* species has been reported to possess an S-layer protein with antibacterial activity.

Previously, the surface layer protein SlpB from *Lactobacillus crispatus* K313 was observed to act synergistically with nisin, resulting in the control of staphylococcal meat decay. However, the mechanism of how SlpB acts in tandem with nisin has not been investigated. A relatively conserved C-terminal region was found between SlpB and SA by sequence alignment, while there was considerable sequence variability in the N-terminus^19^. This suggests that SlpB may have a similar mode of action to SA when combined with nisin against *S. saprophyticus* cells, although further investigation is required. Therefore, our specific objectives were to determine the effect of the combination of SlpB and nisin against the food-borne spoilage bacterium *S. saprophyticus* P2 and clarified how SlpB acted synergistically with nisin.

## Results

### Effects of SlpB and nisin on the growth of *S. saprophyticus*

The minimal inhibitory concentrations (MICs; μg/mL) of nisin and SlpB for *S. saprophyticus* P2 were determined as previously described^20^. The MIC value of nisin was 200 μg/mL, while SlpB delayed the growth of *S. saprophyticus* P2 at 40 μg/mL, however, no inhibition was noted until 300 μg/mL SlpB was utilized. In order to evaluate the effect of the nisin + SlpB combination in *S. saprophyticus* P2, growth curves were determined using cultures treated with either nisin, SlpB, or both agents (Figure S1). Nisin at half of its MIC (100 μg/mL) and SlpB at 40 μg/mL partially delayed the growth at 0–14 h. However, *S. saprophyticus* P2 reached the same cell density as the OD_600_ = 3.2 (5.6 × 10^9^ CFU/mL) with control after 14 h. In contrast, the OD_600_ value of *S. saprophyticus* P2 only reached to 0.5 (7.8 × 10^8^ CFU/mL) after treatment with SlpB and nisin for 14 h. The combination of both SlpB (40 μg/ml) and nisin at a half of MIC (100 μg/mL) inhibited the growth of *S. saprophyticus* P2.

### Lytic activity of SlpB and nisin on *S. saprophyticus* cells

**Figure 1:**
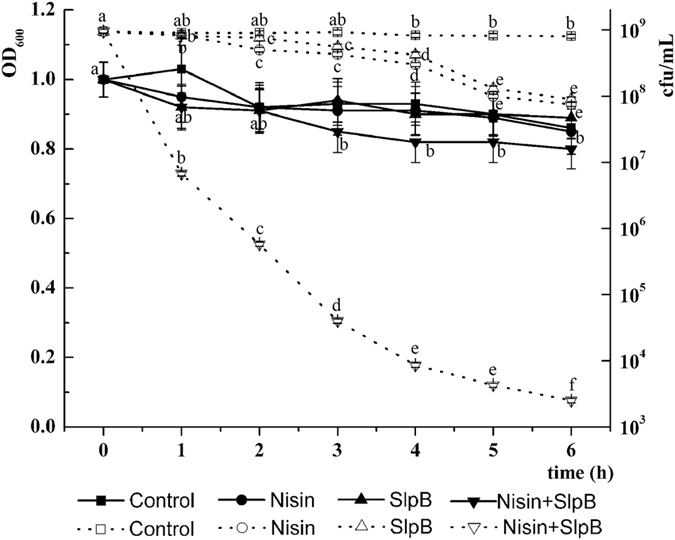
Lytic curves of SlpB, nisin, or nisin + SlpB. Exponentially growing bacteria were incubated in the presence of nisin, SlpB or nisin + SlpB. Nisin concentration, 100 μg/mL; SlpB, 40 μg/mL. Three or more independent experiments were performed. Dotted line, cfu/mL; unbroken line, OD_600_. Different letters in the curve indicate significant difference (p < 0.05).

For analysis of the lytic activity, OD_600_ decrease of exponential cultures of *S. saprophyticus* cells was monitored after exposure to nisin, SlpB or nisin + SlpB. No decrease in cell density was observed in the presence of nisin at 100 μg/mL or SlpB at 40 μg/mL (Fig. 1). A marginal decrease of cell density was observed in the nisin + SlpB treatment (P < 0.05). Neither nisin nor SlpB caused significant lytic activity against *S. saprophyticus* cells. However, this is not consistent with the viable count determination, as a rapid decline in viability was observed even before the OD_600_ decrease that occurred after nisin + SlpB was added. A ~10^5^-fold reduction in viability was noted 6 h after treatment with nisin + SlpB. Thus, cell death caused by nisin + SlpB could not be attributed to cell lysis.

### Effects of SlpB and nisin on *S. saprophyticus* morphology

**Figure 2:**
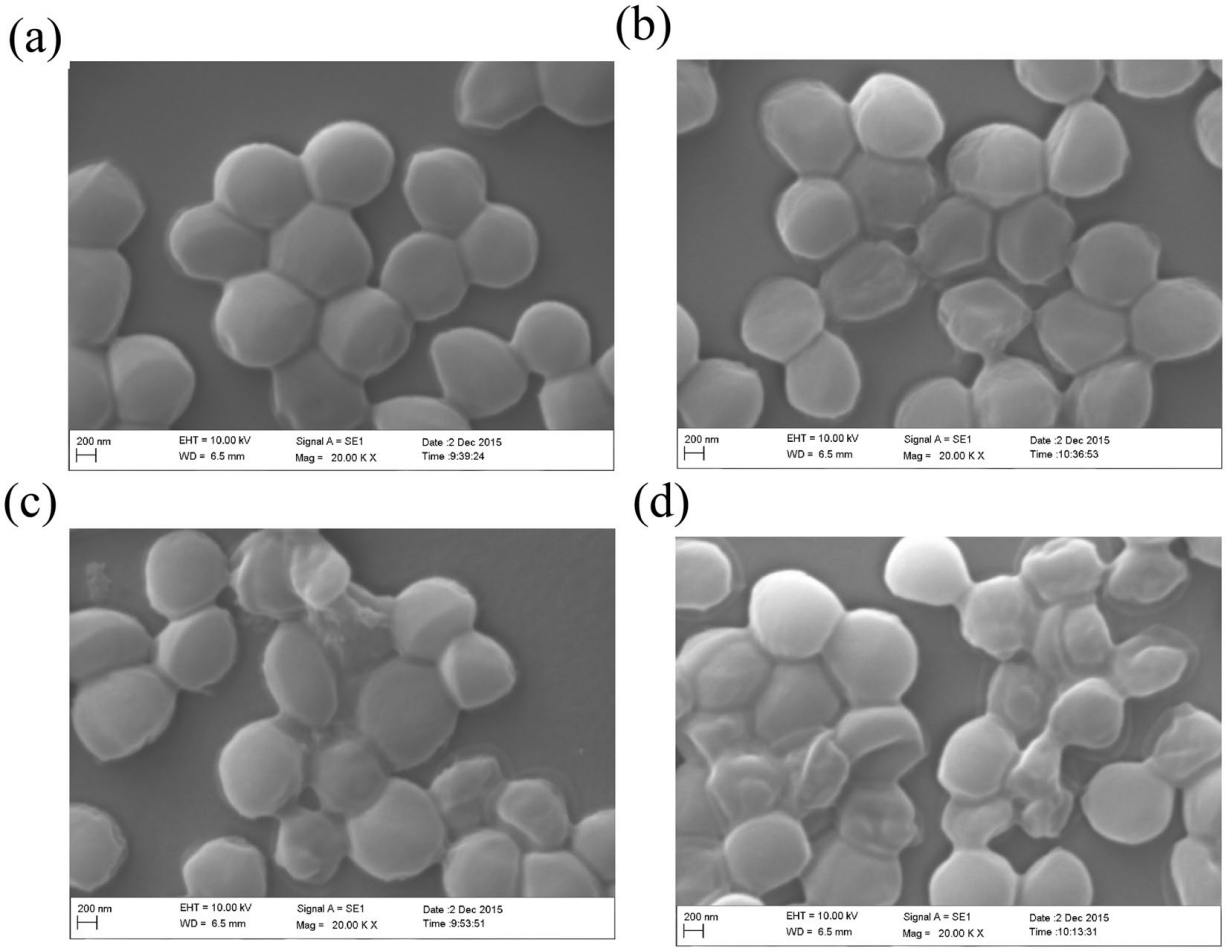
SEM images of untreated *S. saprophyticus* P2 cells (**a**), as well as cells treated with nisin (**b**), SlpB (**c**), and nisin plus SlpB (**d**). Nisin concentration, 100 μg/mL; SlpB concentration, 40 μg/mL.

**Figure 3:**
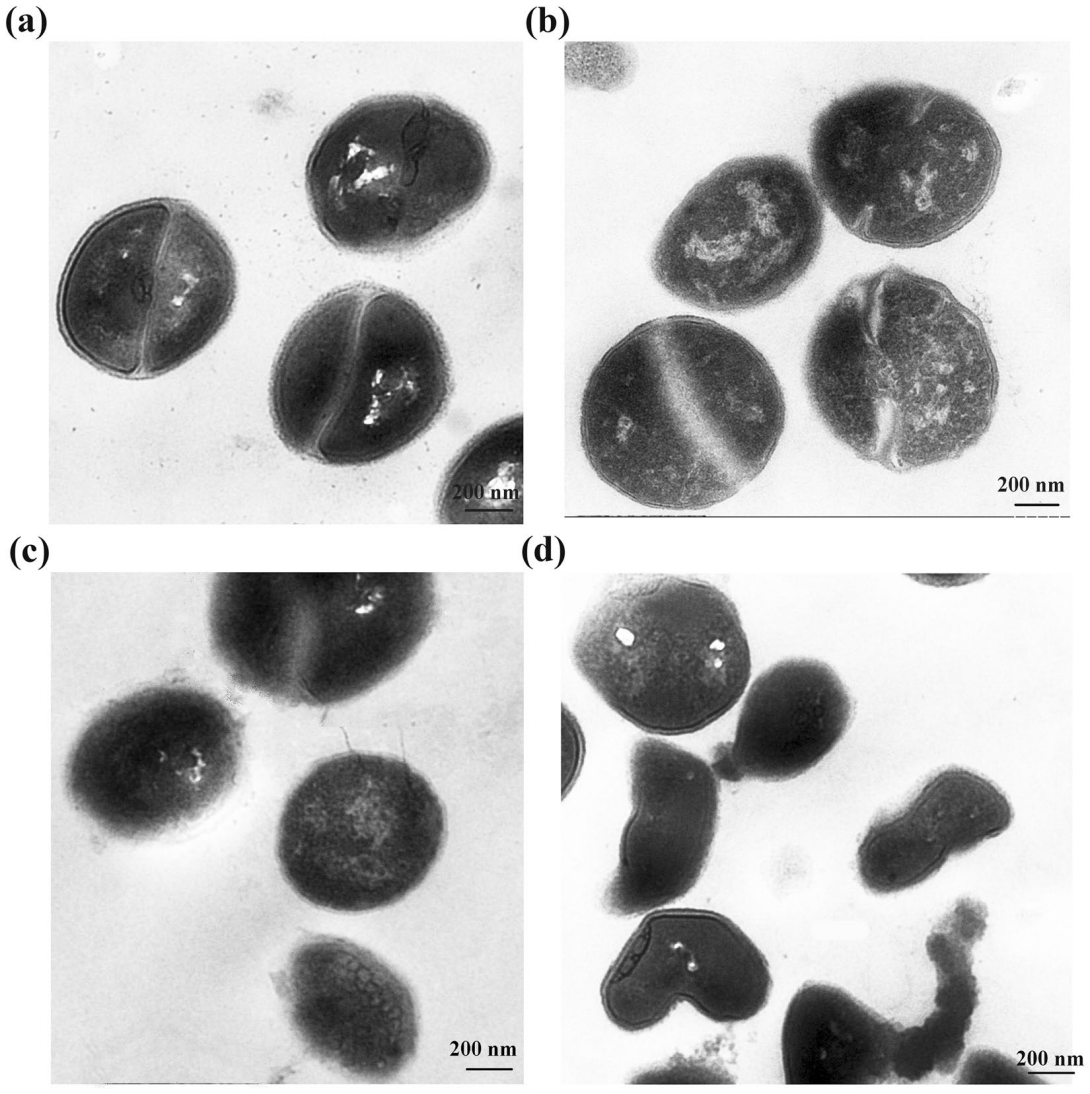
TEM images of untreated *S. saprophyticus* P2 cells (**a**), as well as cells treated with nisin (**b**), SlpB (**c**), and nisin plus SlpB (**d**). Nisin concentration, 100 μg/mL; SlpB concentration, 40 μg/mL.

The morphology of *S. saprophyticus* P2 was determined by scanning electron microscopy (SEM) and transmission electron microscopy (TEM). SEM was used to examine the cell surface, while TEM visualized the intracellular portion of the bacteria. The untreated *S. saprophyticus* P2 displayed intact cell membranes and plump round cells (Fig. 2a). Cells treated with nisin became flat (Fig. 2b), indicating that cytoplasmic material might be released into the extracellular medium. Cells treated with SlpB exhibited slight surface damage (Fig. 2c), while nisin + SlpB treatment caused extensive surface damage to most cells—both a sunken cell surface and shrinking cytoplasm were observed (Fig. 2d). This result indicated that SlpB induced the surface damage of *S. saprophyticus* P2, therefore enhancing the action of nisin and resulting in the significant release of more cytoplasmic materials. TEM images displayed the organization typical for gram-positive bacteria, with clearly defined cytoplasm, plasma membrane (PM) and CW (Fig. 3a). After nisin exposure, no extensive CW damage was observed (Fig. 3b), however SlpB-treated cells demonstrated significant modifications to CW as well as no clear delineation between the CW and the PM (Fig. 3c). After exposure to nisin + SlpB, TEM images revealed the dramatic damage to the PM and CW (Fig. 3d). Considering that nisin is a pore forming bacteriocin on CM and SlpB led to structural damage of the CW, SlpB is speculated to enhance nisin access into cell membrane by enabling travel across the CW.

### Cytoplasmic membrane permeability

#### Measurement of extra- and intracellular ATP

**Figure 4:**
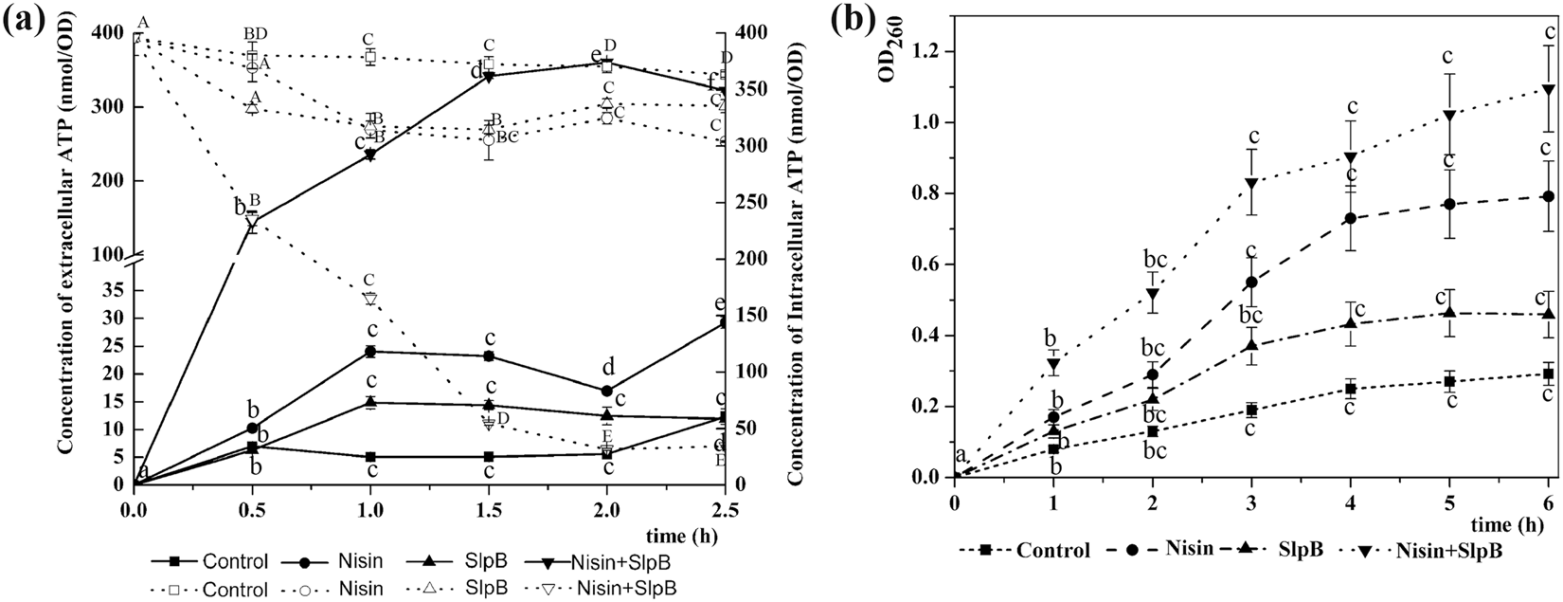
Intracellular and extracellular ATP levels (**a**) and extracellular UV-absorbing materials (**b**) in *S. saprophyticus* P2 cells treated with SlpB, nisin, or nisin + SlpB. Nisin concentration, 100 μg/mL; SlpB concentration, 40 μg/mL. A: Dotted line, intracellular ATP levels; unbroken line, extracellular ATP levels. Different letters in the curve indicate significant difference (p < 0.05).

ATP levels were measured as indexes of cell injury and non-selective pore formation. The extracellular ATP was maintained at ~10 nmol/OD in both SlpB treated and control cells. In contrast, treatment with nisin for 2.5 h resulted in extracellular ATP levels increasing to 29.3 nmol/OD, while nisin + SlpB caused a rapid increase to 144.5 nmol/OD in 0.5 h, which then increased to 322.4 nmol/OD after 2.5 h (Fig. 4a). Conversely, the intracellular ATP levels decreased from 395 to 34.2 nmol/OD within 2.5 h treatment with nisin + SlpB. Measurement of the extra- and intracellular ATP content indicated that treatment with both SlpB and nisin induced massive ATP leakage from the cells, suggesting that synergistic activity of nisin + SlpB may be due to increased cytoplasmic membrane permeability.

#### Measurement of UV-absorbing release materials

The effect of nisin, SlpB and nisin + SlpB on CM permeability was then assessed by measuring released UV-absorbing materials. Nisin + SlpB induced the release of material that absorbed at 260 nm (Fig. 4b), which was interpreted to be mostly DNA, RNA, metabolites and ions^21^. This result implied that the synergistic activity of nisin and SlpB increased cytoplasmic membrane permeability, releasing UV absorbing substances. Different from ATP curves, OD_260_ values significantly increased over time from cells treated with nisin (100 μg/mL) or SlpB (40 μg/mL) (P < 0.05). However, increased absorbance of UV was not so conspicuous compared to ATP leakage in nisin + SlpB treated samples.

#### Confocal laser scanning microscopy (CLSM) analyses

**Figure 5:**
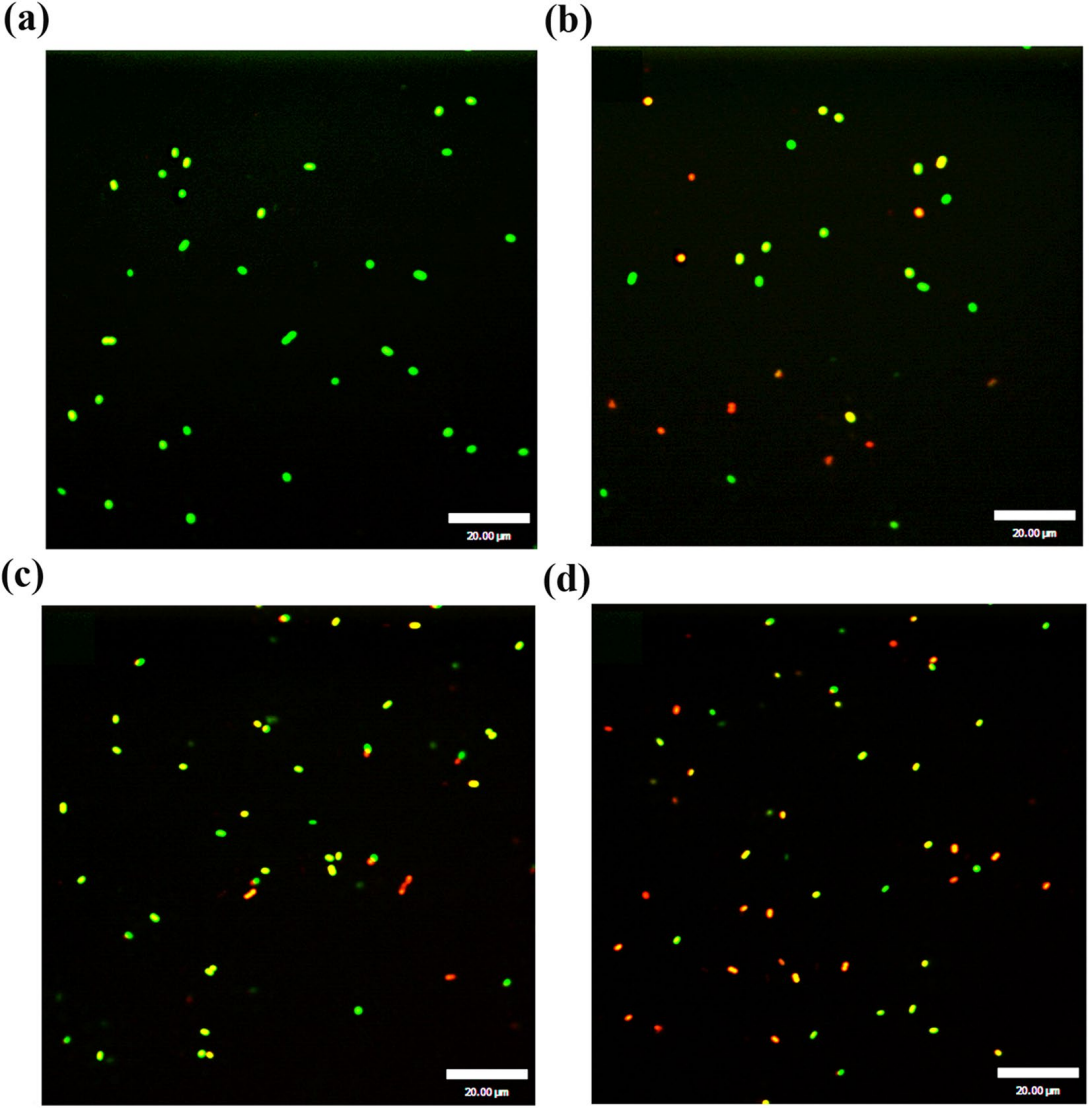
Confocal laser scanning micrographs of untreated *S. saprophyticus* P2 cells (**a**), and cells treated with nisin (**b**), SlpB (**c**), nisin + SlpB (**d**). Nisin concentration, 100 μg/mL; the concentration of SlpB concentration, 40 μg/mL.

The cytoplasmic membrane permeability was also determined by CLSM, using two fluorescent probes, carboxyfluorescein diacetate (cFDA) and propidium iodide (PI), which could distinguish intact from membrane-damaged cells. cFDA, a lipophilic non-fluorescent precursor that readily diffuses across cell membranes, is widely used for the assessment of cellular nonspecific enzymatic activity. Once inside the cell, cFDA is converted by a nonspecific esterase into a polar, membrane-impermeant green fluorescent compound carboxyfluorescein (cF)^22^. PI is a nucleic acid dye, but can enter into cells with compromised membranes and bind to the DNA and RNA, giving a red fluorescence. cFDA generally stains all bacterial cells in a population whereas PI penetrates only when the bacterial membrane is damaged. Thus, bacterial cells with intact membranes will exclude PI, while being stained by cFDA and emitting green fluorescence, whereas bacterial cells with damaged membranes will be stained with PI and emit a red fluorescence. Sub-lethally injured cells that still have esterase activity and compromised membranes will be stained by both PI and cFDA, resulting in yellow fluorescence. Most of the untreated cells had distinct green fluorescence (Fig. 5a), indicating cFDA uptake and PI exclusion. After treatment with nisin (100 μg/mL; Fig. 5b) or SlpB (40 μg/mL; Fig. 5c), yellow and red fluorescence were observed, respectively, indicating that the cytoplasmic membrane of treated cells was damaged. Treatment with SlpB yielded more yellow fluorescence in comparison to nisin-treatment, indicating that SlpB resulted in more sublethally damaged cells. Most cells treated with 100 μg/mL nisin and 40 μg/mL SlpB emitted red or yellow fluorescence, suggesting that that SlpB could synergistically increase cytoplasmic membrane permeability when lower concentrations of nisin were added.

### Flow cytometric (FCM) analysis

**Figure 6:**
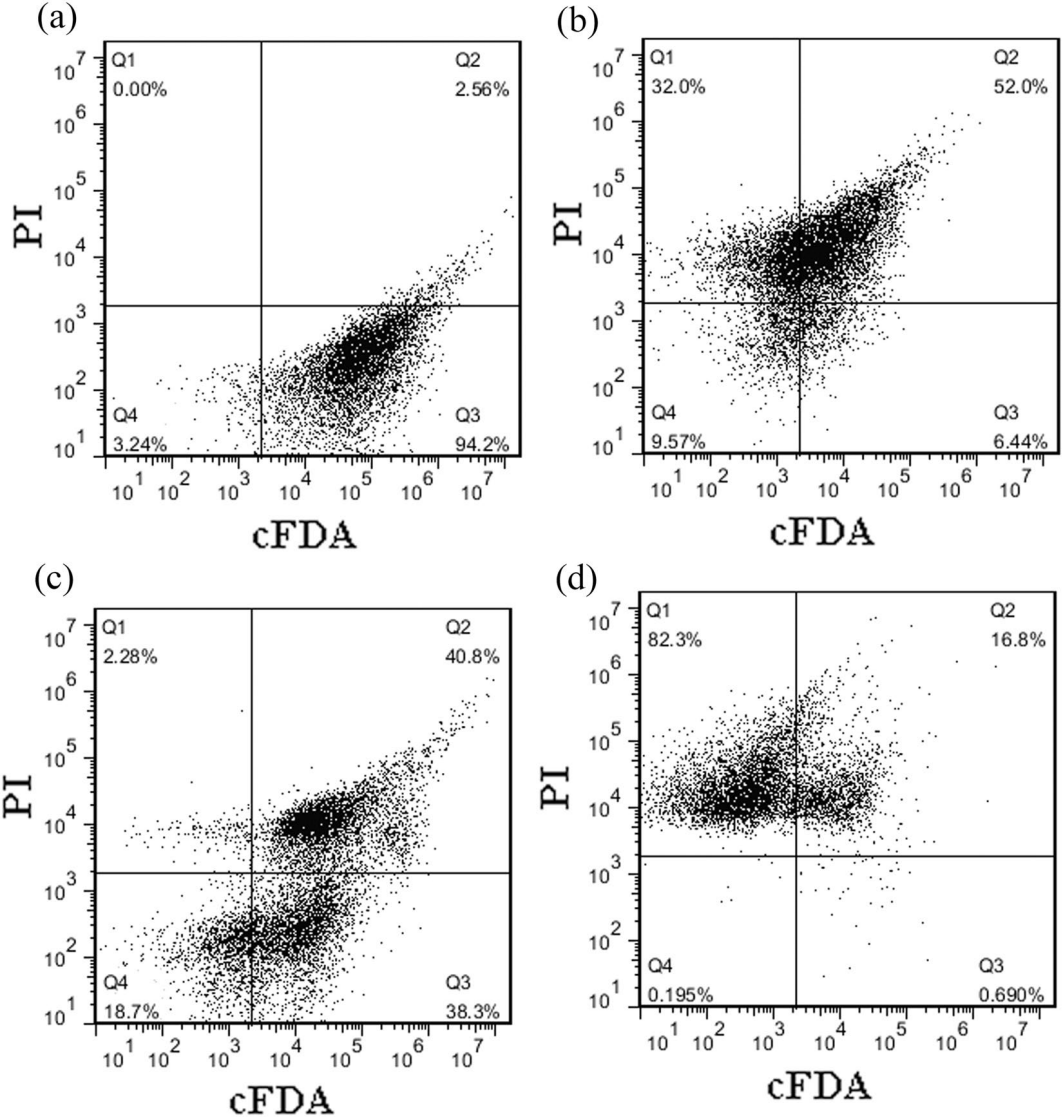
Flow cytometry dot plots of *S. saprophyticus* P2 cells (**a**), and cells treated with nisin (**b**), SlpB (**c**), nisin + SlpB (**d**). Nisin concentration, 100 μg/mL; SlpB concentration, 40 μg/mL.

FCM analysis offers the possibility of physically separating cells by cell sorting for further analysis. PI and cFDA were also chosen to monitor membrane integrity and esterase activity, respectively^22^. The dual-parameter dot plots of *S. saprophyticus* cells stained with cFDA and PI are shown in Fig. 6. 94.2% of the *S. saprophyticus* cells (18,840 events/20,000 events) in the control group were located at the Q3 quadrant (a), indicating that the untreated cells had high esterase activity and intact membranes. After treatment with nisin, the number of intact and sublethal cells distributed in the Q3 and Q2 area was 6.44% and 52.0%, respectively (Fig. 6b). In addition, nearly 32% of the treated cells were gated in Q1, demonstrating that the cell membranes were severely compromised, and the cFDA staining disappeared. After treatment with SlpB, the number of intact cells distributed in the Q3 area was significantly reduced from 94.2% to 38.3% (P < 0.05). Cells distributed in the Q2 region increased from 2.56% to 40.8%, suggesting that the cell membranes were damaged, but the cytosolic enzyme activity persisted with cell in a sublethal state. The results suggested that, after SlpB treatment, the cell membrane was exposed and membrane permeability was increased, while most of the cytosolic enzymes were not affected, indicating sublethal cell injury. In nisin + SlpB-treated cells, the cells distributed in the Q3, Q2, and Q1 region were 0.69%, 16.8%, and 82.3%, respectively. 99.1% of membranes with compromised cells (19,820 events/20,000 events) were observed. It suggested that combination of SlpB and nisin caused serious cell damage and that SlpB could significantly improve the antibacterial effect of nisin.

### Effect of SlpB plus nisin on the transmembrane electrical potential (Δψ)

**Figure 7:**
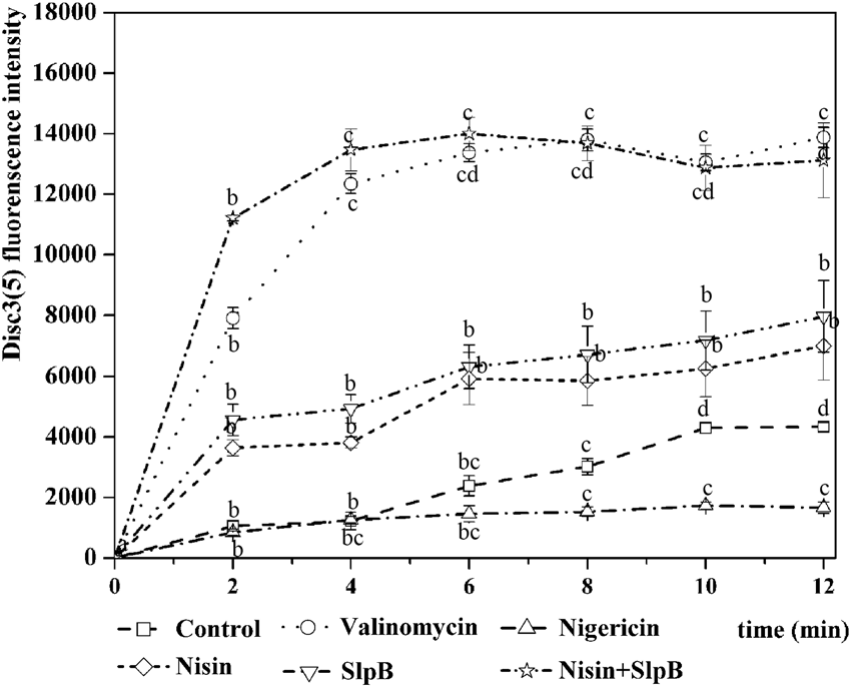
Δψ in *S. saprophyticus* P2 cells. Samples were taken at regular intervals from control cells and following supplementation with nigericin, valinomycin, SlpB, nisin, or nisin + SlpB. Different letters in the curve indicate significant difference (p < 0.05).

In order to further determine the effect of SlpB on the bacterial cytoplasmic membrane, the Δψ of treated cultures was examined (Fig. 7). Treated cells were able to maintain a maximum Δψ, which could be completely dissipated by the addition of the K^+^ ionophore valinomycin (1.0 μM). When the channel forming agent nigericin (1.0 μM) was added, the cells also maintained their Δψ. Twelve min after treatment with SlpB or nisin, the fluorescence values increased to 7962 and 6986, respectively. 40 μg/mL SlpB dissipated ~57.4% of the Δψ, a higher percentage than with nisin (P < 0.05). The combined nisin + SlpB treatment caused an immediate and complete loss of Δψ, indicating that the cytoplasmic membrane was rapidly depolarized. Dissipation of Δψ is a typical characteristic of pore-forming nisin, which suggests a disturbance in cell membrane integrity^23^. These results suggested that SlpB might improve the antibacterial effect of nisin by dissipating Δψ, reflecting pore formation.

ΔpH means transmembrane pH gradient, one component of the proton motive force (PMF). Together with Δψ reflect the changes in PMF. The changes in PMF indicate pore formation and subsequent leakage of essential ions and micromolecules. The dissipation of ΔpH was determined by V,7V-bis-(2-carboxyethyl) 5(and-6)-carboxyfluorescein (BCECF) and no change in the fluorescence was observed (data not shown). This is consistent with a previous report that nisin could not immediately dissipate ΔpH^23^.

### Inhibition of *S. saprophyticus* P2 growth on chicken meat products

The total viable counts (TVC) of chicken meat samples from 5 different treatments are reported in Table 1 as a function of storage time. The initial TVC value of control chicken meat was 2.6 log cfu/g. TVC reached 6 log cfu/g on day 6 for control samples and day 9 for the SlpB- and nisin-treated samples, respectively. The 0.5 g/kg or 0.25 g/kg nisin + SlpB-treated samples were <6 log cfu/g throughout the entire storage period. SlpB alone had a controlling effect on TVC, although to a lesser extent than nisin. Moreover, SlpB significantly improved the efficacy of nisin in controlling microbial growth. Thus, the microbiological shelf life was extended by 6 days using the nisin + SlpB and 0.5 nisin + SlpB treatments.

**Table 1:** The total viable counts in the samples treated with different antibacterial substances during storage.

Storage days (d)	*Staphylococcus saprophyticus* P2 counts (log CFU/g)^a,b^				
	Con	S	N	NA + S	NB + S
0	2.6 ± 0.12A	2.6 ± 0.12A	2.6 ± 0.12A	2.6 ± 0.12A	2.6 ± 0.12A
3	3.27 ± 0.01A	3.16 ± 0.01AC	2.73 ± 0.14B	3.02 ± 0.03C	3.20 ± 0.03AC
6	6.23 ± 0.01A	5.18 ± 0.07B	4.30 ± 0.01B	3.76 ± 0.02C	4.43 ± 0.07BC
9	9.46 ± 0.01A	7.65 ± 0.02B	6.41 ± 0.09C	4.37 ± 0.12D	5.27 ± 0.05CD
12	9.58 ± 0.2 A	8.34 ± 0.06B	6.12 ± 0.13C	4.99 ± 0.03D	5.25 ± 0.02D

^a^Values are averages ± standard deviations. Different uppercase letters in a row indicate significant difference (p < 0.05).

^b^Con: control sample, no SlpB/nisin added; S: SlpB 40 μg/g; N: nisin 0.5 mg/g; NA + S: nisin 0.5 mg/g plus SlpB 40 μg/g; NB + S: nisin 0.25 mg/g plus SlpB 40 μg/g.

### Inhibition of Total volatile basic nitrogen (TVB-N) content on chicken meat products

TVB-N content is an important index of meat freshness. In order to assess the role of nisin and SlpB in this regard, the TVB-N content of samples treated with different antibacterials is shown in Table 2. The TVB-N values of chicken meat samples increased from an initial value of 5.6 mg/100 g meat samples to 30.96, 15.07, 18.37, 10.07 and 15.35 mg/100 g meat samples for the control, and the nisin-, SlpB-, nisin + SlpB-, and 0.5 nisin + SlpB-treated samples, respectively, after 12 days. A TVB-N level of 15–25 mg/100 g meat is considered to indicate a poor freshness, while 25 mg/100 g in chicken meat indicates spoilage^24^. The TVB-N value of untreated samples reached 20.47 and 30.96 mg/100 g chicken meat after 9 and 12 days of storage, respectively, suggested for freshness and spoilage in chicken meats. The TVB-N value was 15.07 or 18.37 mg/100 g meat on day 12 for nisin or SlpB-treated samples, respectively. These values did not reach the limit value of 25 mg/100 g meat, even though the SlpB-treated samples had higher TVB-N values than the nisin-treated samples, which suggests that SlpB alone is less effective in freshness. However, 10.07 and 15.35 mg/100 g meat were observed for nisin + SlpB and 0.5 nisin + SlpB treatments, respectively. Interestingly, the TVB-N values were the same for both full-strength and half-strength nisin treatments. This suggests that SlpB could significantly improve the effect of nisin in maintaining freshness, which was in the agreement with the TVC results.

**Table 2:** TVB contents in the samples treated with different antibacterial substances during storage.

Storage days (d)	TVB-N contents (mg/100 g meat sample)				
	Con	S	N	NA + S	NB + S
0	5.60 ± 0.2A	5.60 ± 0.2A	5.60 ± 0.2A	5.60 ± 0.2A	5.60 ± 0.2A
3	8.82 ± 1.16A	8.52 ± 1.21A	8.23 ± 1.17A	7.93 ± 1.13A	8.58 ± 0.82A
6	13.33 ± 0.95A	9.53 ± 0.77AB	8.72 ± 0.84B	8.54 ± 0.92B	8.84 ± 1.11B
9	20.47 ± 1.16A	17.29 ± 1.13B	14.00 ± 0.55BC	9.95 ± 0.71BC	14.19 ± 0.63C
12	30.96 ± 0.75A	18.37 ± 0.71B	15.07 ± 0.89C	10.07 ± 0.88D	15.35 ± 0.65C

^a^Values are averages ± standard deviations. Different uppercase letters in a row indicate significant difference (p < 0.05).

^b^Con: control sample, no SlpB/nisin added; S: SlpB 40 μg/g; N: nisin 0.5 mg/g; NA + S: nisin 0.5 mg/g plus SlpB 40 μg/g; NB + S: nisin 0.25 mg/g plus SlpB 40 μg/g.

## Discussion

Microbial contamination of foods during processing/storage is a major cause of food-borne illnesses and loss in shelf life^25^. Thus, a number of antimicrobial agents are permitted by regulatory agencies to minimize the deterioration of food quality^26, 27^ but an increased interest in using “natural” additives in the food industry limits the preservatives that can be used^28, 29^, especially as many natural antimicrobials have a limited spectrum of activity and are effective only at very high concentrations^30^. Therefore, the combined utilization of antimicrobial agents with complementary roles can enhance antimicrobial efficacy and reduce the minimum effective dose of antibacterial agents^31^.

The combination of nisin and SlpB inhibited the growth of *S. saprophyticus* P2, and the mode of action was investigated via cell lysis analysis and cell viability counts. A rapid and significant decline in viability was noted, whereas no remarkable cell lysis was observed in the presence of both compounds. The morphology of *S. saprophyticus* P2 cells was further detected by SEM and TEM and no major CW damage was observed in nisin-treated cells. However, significant disruptions were noted inside the CW after nisin + SlpB treatment, thus altering the cell shape. The CW is a vital component of vegetative bacteria, and it is responsible for cell shape and for maintaining the intracellular contents/turgor pressure inside the cell^32^. CW damage often results from enzymatic actions, such as murein hydrolases^33^ or intermolecular forces such as chitosan^34^. Murein hydrolases are able to hydrolyze bacterial CW components and cause cell death^35^; this activity generally causes rapid lysis of bacterial cells. Previous studies have indicated that the S-layer protein SA of *L. acidophilus* causes CW peptidoglycan hydrolysis, therefore enhancing passage of nisin into the cell membrane by enabling it to cross the CW^16, 36^. This action caused the rapid lysis of *Salmonella* cells. Additionally, SA alone cannot hydrolyze the CW of Gram-positive bacteria, but when combined with nisin results in cell lysis^16, 36^. The authors speculated that nisin provides the sudden ion-nonspecific dissipation of the proton motive force required to enhance the S-layer murein hydrolase activity. In this study, cell lysis was not observed with any treatment. This suggested that the synergistic mechanism of nisin + SlpB might be different from SA of *L. acidophilus*, especially as SlpB does not cause bacterial lysis. In addition to cell lysis, CW damage can also be caused by intermolecular forces^34^. Chitosan and its derivatives binds to CW by hydrogen bonding, as well as electrostatic and hydrophobic interactions, allowing it to disturb CW function and exhibit the antibacterial activity^37^. Similarly, S-layer proteins can attach to the CW through hydrogen bonds, as well as electrostatic and hydrophobic interaction^13^. Previously, SlpB was observed to bind to negatively charged CW components through electrostatic interactions^19^. Similar to chitosan, SlpB binding to CW might lead to structural disorganization, which may be correlated to morphological alterations of CW, although alone it is unlikely to cause lysis of *S. saprophyticus* cells. This disorganization enhance nisin access to CM, which could cause the dramatic release of micromolecules from the cell. Consequently, damaged cells lack proper energy production and eventually resulting in cell death. However, this action could not cause the collapse of cell wall. That is why cell lysis was not observed in SlpB + nisin treated samples.

Membrane damage was also observed in SlpB-treated cells with CLSM and FCM. However, whether this damage on CM linked to CW rupture remains to be investigated. Acosta *et al.* and Meng *et al.* postulated that nisin provides the sudden ion-nonspecific dissipation of the proton motive force that is required to enhance S-layer endopeptidase activity^16, 36^. In this study, sudden dissipation of Δψ was observed in the nisin + SlpB treatment, whereas no cell lysis was observed. Nisin assembles to form a stable pore in the cell membrane of target cells, thus inducing Δψ dissipation at a higher concentration than its MIC. This sudden Δψ dissipation after treatment with nisin + SlpB might also be due to SlpB-induced structural disorganization of CW, which enhanced nisin access to form a stable pore.

SlpB had been successfully expressed in *Escherichia coli.* The recombinant SlpB displayed the function of cell wall anchoring and collagen adherence^19^. We compared the antibacterial activity of recombinant SlpB with protein purified from *L. crispatus.* No obvious difference was observed. Thus, recombinant SlpB from *E. coli* can be used for biochemical or functional studies. *E. coli* is one of the most extensively used prokaryotic organisms for the industrial production of proteins of commercial interest, if heterologous proteins do not require complex post translational modifications and are expressed in a soluble form^38^. In this work, SlpB expressed in *E. coli* is a soluble form and was easily purified by Ni- nitrilotriacetic acid (Ni-NTA) affinity chromatography (~1 mg/100 mL). Therefore, this protein could be made in larger quantities for food application.

In summary, we report the first evidence that SlpB binds to the CW through electrostatic interactions, which not only reduces CW integrity in vegetative bacteria and enhances the access of nisin to form a stable pore on CM, but also affects PM permeabilization. These actions cause dramatic release of micromolecules from the cell, therefore damaged cells lack proper energy production and eventually resulting in cell death. These results provide an in-depth explanation for inactivation of bacteria by nisin + SlpB treatment. Additionally, it is important to note that the combined effect of nisin + SlpB resulted not only in growth inhibition of cultures that are present in the initial inoculum but also in the ability to produce cell death in pre-grown cultures, thereby killing pathogen cells. The nisin + SlpB is a potentially new antibacterial combination that could be used in the food industry for food preservation.

## Material and Methods

### Chemicals

Nisin, propidiumiodide (PI), carboxyfluorescein diacetate (cFDA), fluorescent probe 3,3-dipropylthia-dicarbocyanineiodide (DISC3(5)), fluorescent pH indicator 7V-bis-(2-carboxyethyl) 5(and-6)-carboxyfluorescein (BCECF), nigericin, and valinomycin were purchased from Sigma (St. Louis, MO, USA). The ATP detection kit was purchased from Beyotime (Beijing, China). All other chemicals were of the highest analytical grade and were purchased from commercial suppliers.

### Bacterial strains and growth conditions

*L. crispatus* K313 was isolated from chicken intestines and deposited in the China Center for Type Culture Collection (CCTCC AB2011142)^19^. *L. crispatus* K313 was grown in MRS broth (Oxoid, Basingstoke, United Kingdom) at 37 °C without shaking. *S. saprophyticus* P2 was isolated from frozen chicken meat and *E. coli* were grown in Luria-Bertani medium at 37 °C aerobically. When appropriate, ampicillin (100 μg/mL) was added to the broth or the agar.

### Expression and purification of S-layer proteins

The putative S-layer protein gene *SlpB* was PCR amplified from the chromosomal DNA of *L. crispatus* K313 using primer pair SBF (5′GAT*GAATTC*AACTACTAACACTGTTACTAAC3′) and SBR (5′TGT*GTCGAC*GAAGTTTGCCTTCTTAAC3′). The PCR fragment was digested with *Eco*RI and *Sal*I and ligated with *Eco*RI-*Sal*I digested pET-22b, generating the vector pET2201^19^. The resultant plasmid pET2201 was transformed into chemically competent *E. coli* BL21 cells, generating the recombinant strain *E. coli*/pET2201. In order to prepare the purified SlpB protein, *E. coli*/pET2201 was grown at 37 °C aerobically to an OD_600_ of 0.8, and then 0.1 mM isopropyl-β-D-thiogalactopyranoside was added to induce the expression of the His-tagged protein. The His-tagged SlpB protein was purified by Ni-NTA affinity chromatography. The purified protein was dialyzed against 20 mM sodium phosphate (pH 7.0) and quantified using Bradford protein assay.

### Effects of SlpB and nisin on growth of *S. saprophyticus*

The MIC of nisin and SlpB for *S. saprophyticus* P2 were determined as previously described^20^. Next, growth curves of *S. saprophyticus* P2 in the presence of nisin (100 μg/mL, 0.5 MIC), SlpB (40 μg/mL), or both (nisin 100 μg/mL plus SlpB 40 μg/mL) were observed. A 2% inoculum of *S. saprophyticus* P2 (2 × 10^7^ CFU/mL) was added to the cultures and then incubated together at 37 °C in a shaker.

### Lytic activity of SlpB and nisin against *S. saprophyticus* cells

In order to detect the lytic activity of SlpB and nisin against *S. saprophyticus* cells, logarithmic phase cells were collected, washed and resuspended in 20 mM phosphate buffer (PBS, pH 7.0) as live cell substrates. Different antibacterial agents—nisin (100 μg/mL), SlpB (40 μg/mL), or both (nisin 100 μg/mL plus SlpB 40 μg/mL)—were added. The lytic activity was assessed by reduction in turbidity using a spectrophotometer and viable counts were determined using serial decimal dilutions^39^.

### Examination of cell morphology by SEM and TEM

SEM and TEM analyses were performed to explore morphology changes of *S. saprophyticus* P2 treated with nisin (100 μg/mL), SlpB (40 μg/mL), or both (nisin 100 μg/mL plus SlpB 40 μg/mL). *S. saprophyticus* P2 cells at logarithmic phase were harvested by centrifugation, then PBS buffer was added to reach OD_600_ = 1.0 value and antibacterial agents were added. Cells resuspended in PBS buffer were used as the control. After treatment at 37 °C for 2 h, the cells were harvested and washed 3 times with PBS buffer. These cells were then fixed with 2.5% (v/v) glutaraldehyde overnight at 4 °C. After serial dehydration, digital images of the treated and untreated *S. saprophyticus* P2 cells were acquired via SEM (EVO-LS10, Zeiss, Germany) at an accelerating voltage of 20 kV and via TEM (H-7650, Hitachi, Japan) at an operating voltage of 80 kV.

### Cytoplasmic membrane permeability

#### Measurement of extra- and intracellular ATP

Extra- and intracellular ATP levels after treatment with nisin (100 μg/mL), SlpB (40 μg/mL), nisin (100 μg/mL) plus SlpB (40 μg/mL) were determined using an ATP detection kit (Beyotime, China). Luminescence detection was performed using an Infinite 200 PRO microplate reader (Tecan, Switzerland).

#### Measurement of UV-absorbing materials

The release of UV-absorbing materials was measured using a UV–VIS spectrophotometer. In brief, bacterial cultures growing at logarithmic phase were diluted in PBS to 5 × 10^8^ cfu/mL. The 5-mL cultures were supplemented with 100 μg/mL nisin, 40 μg/mL SlpB, or 100 μg/mL nisin plus 40 μg/mL SlpB. Unsupplemented cell suspensions were used as the negative control. The bacterial suspensions were incubated at 37 °C while shaking. The OD_260_ was determined at 0, 1.0, 2.0, 3.0, 4.0, 5.0, and 6.0 h. After treatment, 5.0 mL of each sample was removed at each time-point, centrifuged and the supernatants were filtered through a sterile nitrate cellulose membrane (0.22 μm). The OD_260_ value of the supernatant was measured to observe the amount of extracellular UV-absorbing materials released by cells. All the measurements were done in triplicate using a UV-6100 spectrophotometer (Mapada, Shanghai, China).

#### Confocal laser scanning microscopy analyses

Cytoplasmic membrane permeability was also assessed using CLSM. Bacterial cultures at logarithmic phase were treated as described above. After treatment, the cultures were dyed using two fluorescent probes, cFDA and PI at final concentrations of 50 and 15 μM, respectively. The mixtures were incubated in the dark for 15 min at 37 °C and washed twice with 1 mL sterile PBS buffer (pH 7.0). Cells were examined under a UltraView VoX spinning disk confocal microscope (Perkin Elmer, Waltham Mass, USA) with laser light at 488 nm.

### Flow cytometric analysis

Bacterial cultures at logarithmic phase were treated for 4 h using the same antibacterial agents described previously. The treated cells were incubated with 15 μM PI or 100 μM cFDA. For double-staining with PI and cFDA, treated cells were initially dyed with 100 μM cFDA at 37 °C for 15 min to allow intracellular enzymatic conversion of cFDA into cF. Cells were then centrifuged and washed with PBS to remove excess cFDA. This step was followed by incubation with 15 μM PI for 10 min to allow the labeling of membrane-compromised cells^22^. Cells were then washed to remove excessive fluorescent probes. Stained samples were kept in the dark for no more than 1 h before FCM analysis.

FCM analysis was performed with an Accuri C6 (Becton, Dickinson and Company, New Jersey USA). The green and red fluorescence of each cell was measured, amplified, and converted into digital signals for further analysis. cF emits green fluorescence at 525 nm following excitation with laser light at 488 nm, whereas red fluorescence at 620 nm is emitted by PI stained cells. All registered signals were logarithmically amplified. Data acquisition was set to 20,000 events at a low flow rate (400–600 events/s).

### Measurement of the Δψ

Δψ was determined with the fluorescent probe DISC3(5) as previously reported^40^. Exponential *S. saprophyticus* cells were prepared as described above. Cells were suspended in 50 mM potassium HEPES buffer (pH 7.0) supplemented with 10 mM glucose and dyed with 0.5 μM DISC3(5). The cell suspensions were then supplemented with nigericin (1 μM), valinomycin (1 μM), and nisin (100 μg/mL), SlpB (40 μg/mL), or both (nisin 100 μg/mL plus SlpB 40 μg/mL). A cell suspension without any supplementation was used as the control. An Infinite 200 PRO microplate reader (Tecan, Switzerland) was used to detect the fluorescence, using at excitation and emission wavelengths of 622 and 670 nm, respectively. ΔpH was measured by treating the cell suspension—supplemented with 10 mM glucose—with the fluorescent pH indicator BCECF as previously described^23^.

### Analysis of the synergistic antibacterial effect of SlpB and nisin in meat products

*S. saprophyticus* P2 was cultured in 100 mL of Luria Bertani medium at 37 °C with shaking to OD_600_ = 1. Cells were pelleted via centrifugation and then resuspended in sterile normal saline (9 g/L NaCl) and the bacterial samples were diluted to ~10^4^ CFU/mL. Fresh chicken breast meat was diced and the minced meat was then sterilized by irradiation^41^. Afterward, 20 g of minced chicken meat was mixed with 1 mL of diluted *S. saprophyticus* P2 samples with initial bacterial counts of 10^2^–10^3^ CFU/g. The mixed sample was then supplemented with different antibacterial agents as follows: N (0.5 mg/g nisin); S (40 µg/g SlpB); NA + S (0.5 mg/g nisin plus 40 µg/g SlpB); NB + S (0.25 mg/g nisin plus 40 µg/g SlpB). The sample without supplementation was used as a control. All chicken meat samples were packed with aluminum foil pouches in a sterile environment. Storage temperature of fresh meat is usually 4–10 °C^42^. In our text, the pouches were heat sealed using a DZ-600/4 s vacuum packing machine (Nanjing, China) and stored in a refrigerator at 7 ± 0.5 °C according to Aymerich *et al.* ^43^. Sampling was performed at 0, 3, 6, 9, and 12 days. TVB-N was determined according to the method proposed in GB/T 5009.44–1996 (Ministry of Health of the P.R. China, 1996). TVC of *S. saprophyticus* P2 in the samples during storage was obtained using the gradient dilution method previously described. Every treatment was performed in triplicate.

### Statistical analysis of the data

The differences between three groups were evaluated by one-way analysis of variance, using SPSS software and the Pearson correlation coefficient option (version18.0, IBM-SPSS Inc., Armonk, NY)^44^. Differences were regarded as significant at p < 0.05. All data were expressed as mean ± standard error.

## Electronic supplementary material

Supplementary informations
