# Supplementary material for: Synergistic antibacterial mechanism of the Lactobacillus crispatus surface layer protein and nisin on Staphylococcus saprophyticus
Source: Sci Rep. 2017 Mar 21;7:265. doi: 10.1038/s41598-017-00303-8 (PMC5428217; doi:10.1038/s41598-017-00303-8)
Supplement: Supplementary file 1 — Supplementary informations [file 41598_2017_303_MOESM1_ESM.pdf]

Title: Synergistic antibacterial mechanism of the *Lactobacillus crispatus* surface layer protein and nisin on *Staphylococcus saprophyticus*

Authors: Zhilan Sun[a#], Pengpeng Li[a#], Fang Liu[a*], Huan Bian[a], Daoying Wang[a,b*], Xiaomeng Wang[a], Ye Zou[a], Chong Sun[a], Weimin Xu[a,b].

[a] Institute of Agricultural Products Processing, Jiangsu Academy of Agricultural Sciences, Nanjing 210014, PR China

[b] Key Laboratory of Meat Processing and Quality Control, Ministry of Education, Nanjing Agricultural University, Nanjing 210095, PR China

*Corresponding author, Tel.: +86 25 84390065; Fax: +86 25 84395939

E-mail: fangliu82@163.com; daoyingwang@yahoo.com.

[#]Zhilan Sun and Pengpeng Li contributed equally to this article.

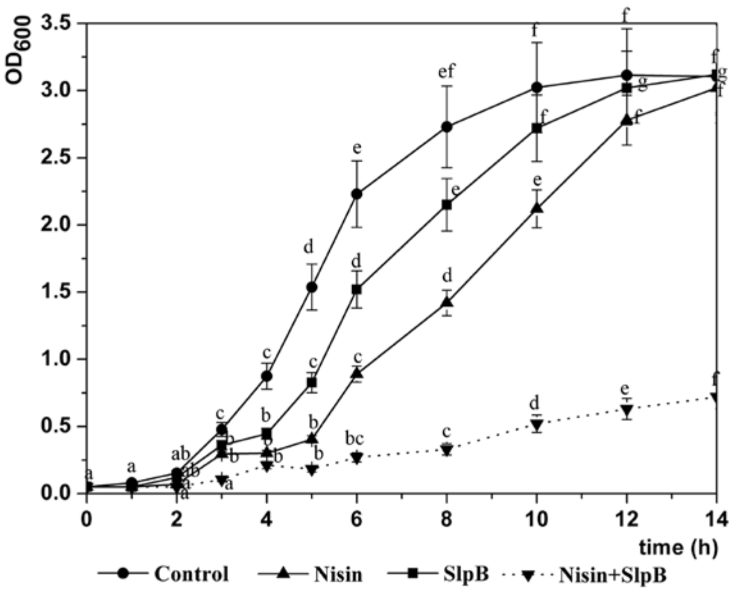

Supplementary Figure S1. Effect of SlpB, nisin, or both on the growth of *S. saprophyticus*. 2% inoculum of *S. saprophyticus* P2 was used in the samples, each containing either the S-layer, nisin, or both, and OD_{600} was monitored every hour. Nisin was added at 100 μg/mL; the concentration of S-layer was 40 μg/mL. Three or more independent experiments were performed for each treatment.
